# Depicting the proton relay network in human aromatase: New insights into the role of the alcohol‐acid pair

**DOI:** 10.1002/pro.4389

**Published:** 2022-08-11

**Authors:** Chao Zhang, Gianfranco Gilardi, Giovanna Di Nardo

**Affiliations:** ^1^ Department of Life Sciences and Systems Biology University of Turin Turin 10123 Italy

**Keywords:** alcohol‐acid pair, aromatase, compound I, cytochromes P450, proton delivery

## Abstract

Human aromatase is the cytochrome P450 catalyzing the conversion of androgens into estrogens in a three steps reaction essential to maintain steroid hormones balance. Here we report the capture and spectroscopic characterization of its compound I (Cpd I), the main reactive species in cytochromes P450. The typical spectroscopic transitions indicating the formation of Cpd I are detected within 0.8 s when mixing aromatase with *meta*‐chloroperoxybenzoic acid. The estrogen product is obtained from the same reaction mixture, demonstrating the involvement of Cpd I in aromatization reaction. Site‐directed mutagenesis is applied to the acid‐alcohol pair D309 and T310 and to R192, predicted to be part of the proton relay network. Mutants D309N and R192Q do not lead to Cpd I with an associated loss of activity, confirming that these residues are involved in proton delivery for Cpd I generation. Cpd I is captured for T310A mutant and shows 2.9‐ and 4.4‐fold faster rates of formation and decay, respectively, compared to wild‐type (WT). However, its activity is lower than the WT and a larger amount of H_2_O_2_ is produced during catalysis, indicating that T310 has an essential role in proton gating for generation of Cpd 0 and Cpd I and for their stabilization. The data provide new evidences on the role of threonine belonging to the conserved “acid‐alcohol” pair and known to be crucial for oxygen activation in cytochromes P450.

## 1 INTRODUCTION

Cytochromes P450 (P450s or CYPs) are heme‐thiolate monooxygenases widely distributed in nature and catalyze various oxidation reactions, including hydroxylation, epoxidation, and heteroatom oxidation,^1^, ^2^, ^3^ thus having some important physiological functions, such as steroids hormone and secondary metabolites biosynthesis, drug metabolism and xenobiotics detoxification.^4^, ^5^, ^6^ Among them, aromatase (CYP19A1) is a well‐conserved enzyme among all vertebrates,^7^ where it is responsible for the transformation of androgens into estrogens, thus playing a crucial role in regulating human sex steroids balance.^8^, ^9^, ^10^, ^11^ This enzyme has received considerable attention because the alteration of its activity, that can also be caused by exogenous substances such as endocrine disrupting chemicals, can affect the endocrine system leading to different pathologies.^12^, ^13^, ^14^ Furthermore, aromatase is an effective target for estrogen‐dependent cancer therapy.^15^, ^16^, ^17^, ^18^

**SCHEME 1:**
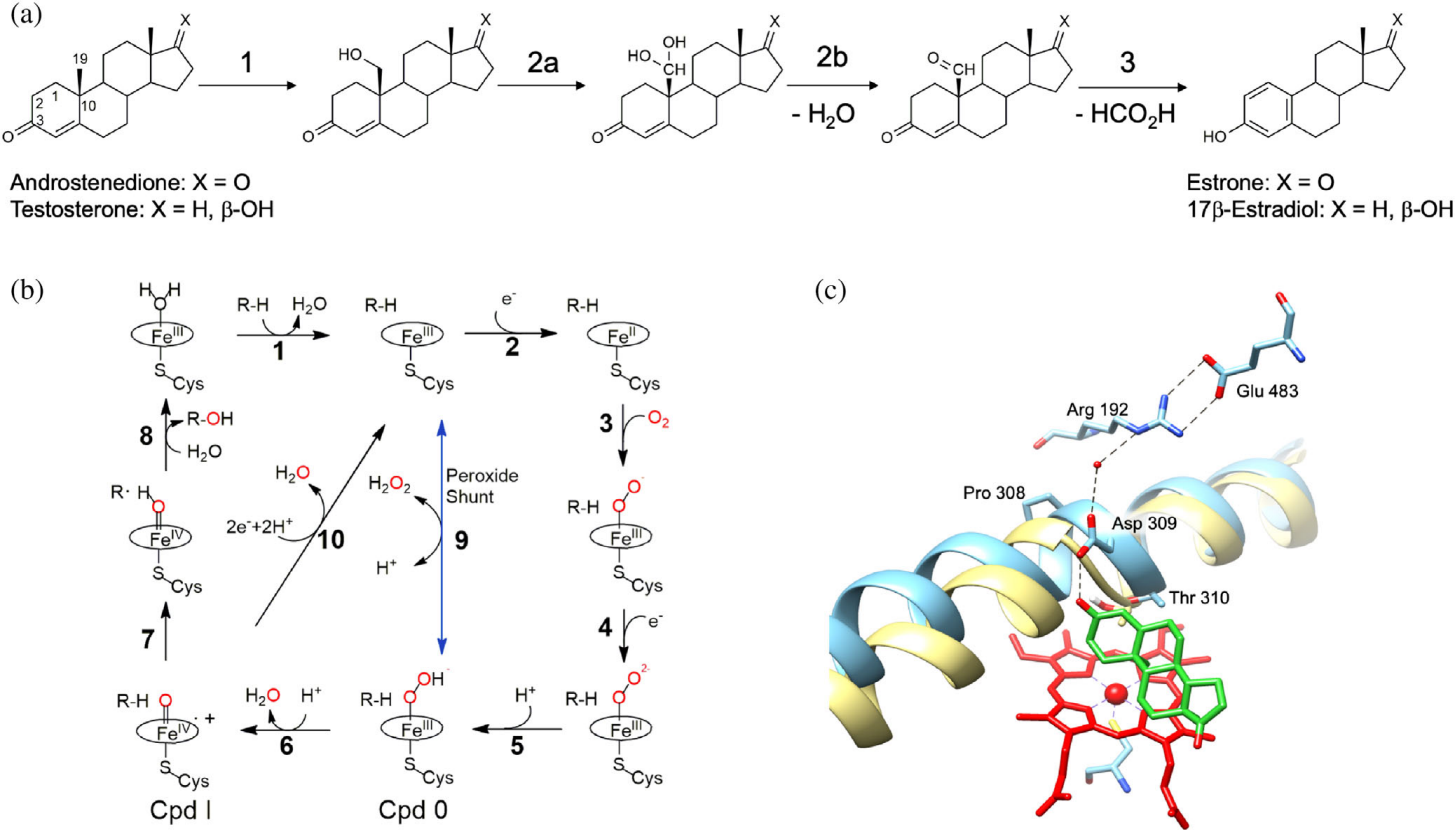
Reaction and catalytic mechanism of human aromatase. (a) The three‐step reaction catalyzed by aromatase allowing the conversion of androgens into estrogens. (b) Catalytic cycle of cytochromes P450 and possible uncoupling pathways. The peroxide shunt pathway can generate directly Cpd 0 (9) and Cpd I (6). (c) Active site of human aromatase (cyan, PDB ID 3S79) with the predicted proton relay network. The helix I is superimposed to the corresponding one in cytochrome P450cam (yellow, PDB ID 5CP4) to show the distortion in human aromatase due to the presence of Pro308. The heme is shown in red and the substrate androstenedione in green. Dashed black lines represent the predicted proton relay network.

From the catalytic reaction perspective, aromatase is a very intriguing enzyme as it is one of the few enzymes that can generate aromatic rings. The aromatization reaction requires three sequential steps where the 19‐methyl group of the androgen is first hydroxylated at C19, then again hydroxylated to produce a 19‐gem‐diol intermediate that spontaneously dehydrates to form an aldehyde (Scheme 1a).^19^, ^20^ In the third step, oxidative cleavage occurs on the carbon bond between C19 and C10, which results in the release of C19 as formic acid, accompanied by the A ring aromatization of the steroid (Scheme 1a).^21^, ^22^

A close look at the general P450 catalytic cycle shows that there are two protonation steps in the formation of Compound I (Cpd I) (Scheme 1b): in the first step, the peroxo‐ferric intermediate is protonated at the distal oxygen to form the hydroperoxo‐ferric complex, also known as Cpd 0 (Scheme 1b, 5); in the second one, the Cpd 0 receives another proton and the following cleavage of the O–O bond forms Cpd I (Scheme 1b, 6).^23^ After the first step, if the coordinated peroxide dissociates, it leads to “uncoupling” and produces hydrogen peroxide (Scheme 1b, 9). After the second protonation step, another uncoupling reaction can take place from Cpd I producing a water molecule (Scheme 1b, 10).

By analogy and comparison with other P450 catalyzed reactions, it is widely accepted that the first and second oxidation steps catalyzed by aromatase are classic hydroxylation mediated by the active iron species Cpd I (Scheme 1b, 7).^24^, ^25^, ^26^ However, the identity of the intermediate involved in the third step (Cpd 0 vs. Cpd I) has been controversial for many years and only recent experimental results suggested that the last step is still mediated by Cpd I.^27^, ^28^, ^29^

It is known that the well‐conserved acid‐alcohol pair in cytochromes P450, located on helix I, is involved in oxygen activation to generate Cpd I.^23^, ^30^ In particular, the acidic residue is part of the proton relay network. Mutation of this residue in different cytochromes P450 leads to a significant reduction of the NAD(P)H oxidation rate and the enzyme activity.^31^, ^32^, ^33^, ^34^, ^35^ The role of the threonine belonging to this acid‐alcohol pair is more intriguing because its mutation into an alanine has a different effect on various P450 enzymes. In P450cam, the mutant D251N shows a small reduction in NADH consumption, but the enzyme becomes 95% uncoupled.^36^, ^37^ The crystal structure of the mutant suggested a role of this residue as a hydrogen bond acceptor for Cpd 0 with a consequent stabilization of this intermediate that promotes the second proton transfer to form Cpd I.^38^

However, the mutation of the Thr belonging to the acid‐alcohol pair in other P450 enzymes has shown that this residue is important but not essential for catalysis.^39^ For example, in cytochrome P450 BM3, the activity of the mutant T268A was shown to depend on the substrate used, and for some substrates, it was possible to obtain the same activity as the wild‐type (WT) protein.^40^ More recently, the T252A mutant of the bacterial CYP199A4, showed a reduction of the activity and coupling efficiency not as dramatic as in P450cam.^39^ For P450cam, CYP199A4 and P450 BM3, the crystal structures of the WT proteins and their Thr‐lacking mutants, have shown significant differences providing the structural basis for the different effects of Thr mutation. Indeed, in P450cam, the crystal structure of WT and T252A mutants in complex with dioxygen is also available and shows that, upon oxygen binding, there is a change in helix I conformation and a flip of the aspartic acid residue by 90° (D251). This allows access into the active site of two water molecules that take part to a hydrogen bond network proposed to enable proton delivery.^38^, ^41^ An enlargement of the oxygen‐binding groove is also present in the structure of P450cam T252A mutant compared to that of WT.^42^ In CYP199A4 and P450 BM3, such structural changes on helix I that modify the oxygen binding groove are not as large as in P450cam in the corresponding threonine mutants.^39^, ^43^ In these enzymes, the most significant differences compared to P450cam were found to be the presence of a second Thr residue that contributes to the hydrogen bonding network and the lack of a salt bridge between the acidic residue of the acid‐alcohol pair and a positively charged one.^39^

In aromatase, an overall different situation is present. The crystal structure in complex with the substrate showed that the aspartate belonging to the acid‐alcohol pair (D309) is not involved in a salt bridge as in P450cam and it forms a hydrogen bond with the 3‐keto group of the substrate androstenedione (ASD) that is predicted to accept the proton from D309 for substrate aromatization.^44^, ^45^, ^46^ Besides, D309 is also connected through a water molecule to an arginine residue (R192) that forms a salt bridge with E483 (Scheme 1c).^44^, ^47^ Thus, D309 and R192, together with a water molecule, are predicted to form the proton relay network.^44^, ^47^ Indeed, their mutation in non‐protonable residues resulted in a significant decrease in activity.^35^ Furthermore, instead of a second threonine after T310 belonging to the acid‐alcohol pair as found in CYP199A4 and P450 BM3, there is a methionine residue (valine in P450cam). This leads to questions as to whether D309 in aromatase plays a double role (proton delivery for oxygen activation and proton delivery to the substrate), and whether the T310 is essential for catalysis.

Here, we report the capture and characterization of Cpd I in human aromatase and we provide direct evidence for its involvement in the last aromatization step of reaction. Moreover, we investigate by site‐directed mutagenesis the role and involvement of D309, T310, and R192 in Cpd I formation and catalysis. Since the acid‐alcohol pair is a well‐conserved motif in cytochromes P450, with some exception represented, for example, by P450 acting as peroxygenases,^48^ P450cin^31^ and P450eryF,^49^ the data obtained for aromatase show significant differences with the other bacterial cytochrome P450 enzymes characterized, providing new insights in P450 mechanism of oxygen activation.

## 2 RESULTS

### 2.1 Compound I in aromatase: Capture and characterization

**FIGURE 1:**
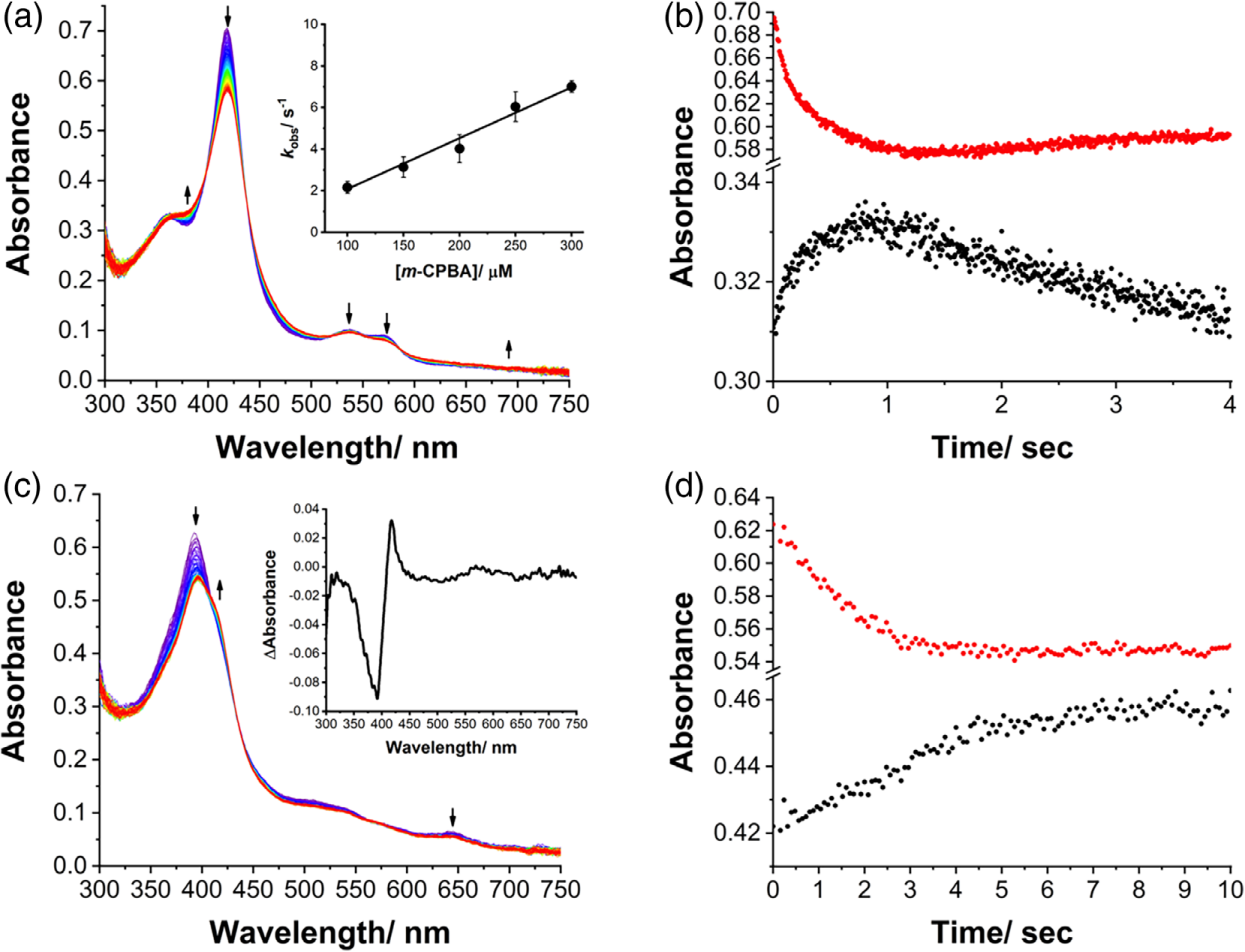
Compound I formation in aromatase WT. (a) UV/Visible spectral changes in 0.8 s after mixing of 7 μM aromatase WT (final concentration) with 200 μM *m*‐CPBA (final concentration) in 100 mM potassium phosphate buffer, 10% glycerol, pH 7.5, at 4°C. Inset: plot of observed rate constants (*k*_obs_) for Cpd I formation against various concentrations of *m*‐CPBA at pH 7.5 and 4°C. (b) Kinetic traces of reaction between aromatase WT and *m*‐CPBA at 380 nm (black circles) and 418 nm (red circles) in 4.0 s. (c) Spectral changes observed upon mixing 7 μM aromatase (final concentration) in complex with the 19‐oxoandrostenedione with 200 μM *m*‐CPBA (final concentration) in 100 mM potassium phosphate buffer, 10% glycerol, pH 7.5, at 4°C. Inset: Difference spectrum obtained by subtracting the spectrum recorded after 4 s and the initial one. (d) Kinetic traces of reaction between aromatase WT in complex with the 19‐oxoandrostenedione and *m*‐CPBA at 380 nm (black circles) and 418 nm (red circles) in 4.0 s. WT, wild‐type.

The principal reactive intermediate involved in catalysis in cytochromes P450 (Cpd I) has been spectroscopically captured and characterized in different P450 enzymes mainly from bacteria^26^, ^50^, ^51^, ^52^, ^53^ and the mammalian rabbit P450 2B4.^54^ This has been achieved through the peroxide shunt obtained by mixing the enzyme with oxidizing agents such as *meta*‐chloroperoxybenzoic acid (*m*‐CPBA) and transiently detecting its absorbance spectrum using stopped‐flow techniques. The same approach is used in this work for a recombinant form of human aromatase^55^ that will be referred to as aromatase WT. The data in Figure 1a show the spectral changes obtained upon mixing 7 μM of aromatase (WT) with 200 μM of *m*‐CPBA in 0.8 s at 4°C. A rapid decrease in the Soret region at 418 nm and a concurrent increase of absorption at 380 nm is observed. Moreover, the two bands at 537 and 570 nm decrease and a band at 610 nm appears. These spectral features are consistent with the ones previously observed for Cpd I in other P450 enzymes.^26^, ^50^, ^51^, ^52^, ^53^, ^54^ We do not detect the band at 690 nm previously observed with the lowest absorption in CYP119 due to the lower yield of Cpd I formation in aromatase WT. However, the spectral changes observed with five isosbestic points at 353, 399, 435, 523, and 582 nm are consistent with previous reports that indicate the formation of Cpd I.^26^, ^53^

Kinetic experiments of the reaction between aromatase and *m*‐CPBA at 380 and 418 nm (Figure 1b) show that Cpd I forms in 0.8 s and decays in 4 s. Figure 1b shows a first exponential increase at 380 nm, followed by a linear decrease that reflects the formation and decomposition of Cpd I,^51^, ^56^ respectively, that can be described by Equation (1):

(1) $$\;\text{Aromatase}\;\left(\text{ferric}\right)\overset{k_{1}}{\underset{⇢\mathrm{Cpd}\;0\to }{\to }}\text{Cpd I}\;\overset{k_{2}}{\to }\text{Aromatase}\;\left(\text{ferric}\right)$$

In Equation (1), *k*_1_ and *k*_2_ are formation and decomposition rate constants of Cpd I, respectively. The absorbance at 418 nm shows an opposite trend as a function of time. To estimate the observed rate constant (*k*_obs_) different amounts of *m*‐CPBA are tested and different *k*_1_ values are obtained and plotted as a function of *m*‐CPBA concentration (inset in Figure 1a). A linear trend is obtained, and the slope of linear fitting provides a second‐order rate constant of 0.25 (± 0.01) × 10^5^ M^−1^ s^−1^. The decomposition of Cpd I follows a first‐order kinetic process and is independent of the concentration of *m*‐CPBA.^51^ The *k*_2_ can be obtained directly by exponential equation fitting, which is 0.16 (± 0.01) s^−1^.

To confirm the capture of the reactive species Cpd I in aromatase WT, the same experiment is performed with the enzyme in the presence of the substrate. Since the last step of aromatase reaction has been the focus of many studies aimed at elucidating which reactive species (Cpd 0 or Cpd I) is involved in this crucial aromatization reaction, we investigate the aromatization step using the last intermediate 19‐oxoandrostenedione (19‐oxo ASD) as substrate.

Figure 1c shows the spectral transitions observed by mixing aromatase in complex with 19‐oxo ASD and *m*‐CPBA in 4 s. A red‐shift is observed for the Soret peak, with a decrease of the band at 394 nm, typical of the high spin state of Aro in complex with the substrate, and an increase at 418 nm, typical of the low spin substrate‐free aromatase (inset Figure 1c). These data are interpreted with substrate conversion into the product that is released from the active site. The lack of a complete shift from 394 to 418 nm is consistent with an insufficient Cpd I amount for the total conversion of the substrate. The kinetic traces at 418 and 394 nm show that the spectral changes occur within 4 s, the timescale where Cpd I formation and decay were previously found (Figure 1d).

To give further evidence for Cpd I formation and for its involvement in the aromatization reaction, different reactions are set up by adding the substrate 19‐oxo ASD after 1 s from the addition of *m*‐CPBA. The reactions are then pooled together to check the presence of estrone that is detected by HPLC (Figure S1). This result also supports Cpd I as the reactive species for aromatization reaction. Several studies suggested that the ferric‐peroxyanion gives a nucleophilic attack of the Fe(III)–O–O^−^on the acyl carbon to furnish a tetrahedral intermediate which fragments, leading to acyl–carbon cleavage.^57^ However, activity assays in the presence of ^18^O_2_ in combination with high‐resolution mass spectrometry, resonance Raman spectroscopy studies and the evaluation of solvent isotope effect in steady‐state turnover of aromatase, suggested that Cpd I is the active iron species rather than ferric peroxide.^27^, ^28^, ^29^ Our results add direct evidence that this compound mediates the aromatization process.

### 2.2 Role of D309, T310, and R192 in Cpd I formation

The role of the amino acids of the acid‐alcohol pair (D309 and T310) and R192 in Cpd I formation is investigated by site‐directed mutagenesis. To this end, the mutants are expressed and purified in the absence of substrates or inhibitors. The UV–Vis spectrum of the D309N mutant in the resting state shows a maximum absorption peak at 423 nm and a small shoulder peak near 394 nm (Figure S2a) indicating that the mutant is partially in a high spin state even in the absence of any ligand. When it is mixed with *m*‐CPBA, the absorption at 423 nm shows an increase followed by a decrease within 4 s. The same phenomenon occurs at 540 and 570 nm. However, the absorption at 394 nm decreases over time, and the same behavior is observed near the peak at 640 nm. Thus, the typical transitions associated to Cpd I formation are not detected for D309N mutant. The mutant is then co‐purified with the substrate 19‐oxo ASD and shows the same maximum absorption at 394 nm as WT in complex with the substrate. When D309N in complex with the substrate androstenedione is mixed with *m*‐CPBA, no spin shift is observed, even if the peroxide concentration is increased or the reaction time is prolonged (Figure S2b). Such a shift from 394 to 418 nm was previously observed in WT, indicating the progress of the catalytic oxidation of the substrate (Figure 1c). These data are consistent with the activity reported for D309N mutant that is decreased by 98% compared to WT.^35^

The spectra of R192Q reacting with *m*‐CPBA only decrease in the Soret region and do not show the typical transitions related to Cpd I formation (Figure S2c). In addition, no red shift was observed in the reaction with the substrate‐bound R192Q (Figure S2d). The replacement of Arg192 with Gln significantly affects the formation of Cpd I, consistent with the 88% reduced activity of this mutant compared to WT.^35^

Several explanations can be offered to interpret why Cpd I is not detected in mutants D309N and R192Q. These two residues are part of the proton delivery network. The mutation of these two sites interferes with the proton transfer pathway and makes protons unable to be transferred. Another possible reason is that these mutations change the structure near the heme pocket, and change the position of water molecules, which may reduce the availability of protons required for heterolysis and formation of Cpd I, limiting or preventing its formation in the mutants.

**FIGURE 2:**
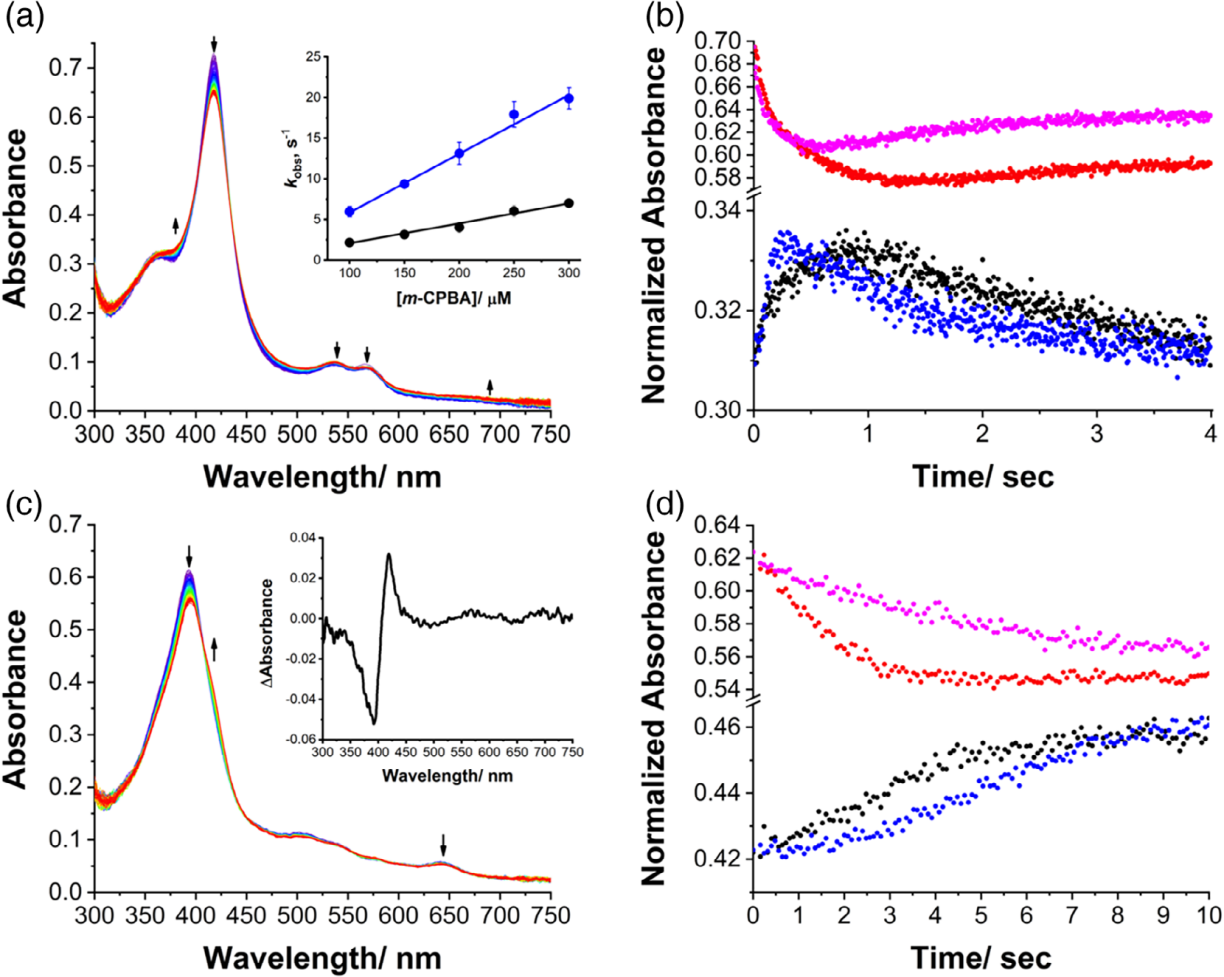
Compound I formation in aromatase mutant T310A. (a) UV/Visible spectral changes in 0.8 s after mixing of 7 μM T310A (final concentration) with 200 μM *m*‐CPBA (final concentration) in 100 mM potassium phosphate buffer, 10% glycerol, pH 7.5, at 4°C. Inset: plot of observed rate constants (*k*_obs_) for Cpd I formation against various concentrations of *m*‐CPBA at pH 7.5 and 4°C for T310A (blue circles) and WT (black circles). (b) Kinetic traces of reaction between T310A and *m*‐CPBA at 380 nm (blue circles) and 418 nm (magenta circles) in 4.0 s. For comparison, the traces at 380 nm (black circles) and 418 nm (red circles) for WT are also shown. (c) Spectral changes observed upon mixing 7 μM T310A (final concentration) in complex with the 19‐oxoandrostenedione with 200 μM *m*‐CPBA (final concentration) in 100 mM potassium phosphate buffer, 10% glycerol, pH 7.5, at 4°C. Inset: Difference spectrum obtained by subtracting the spectrum recorded after 4 s and the initial one. (d) Kinetic traces of reaction between T310A in complex with 19‐oxoandrostenedione and *m*‐CPBA at 418 nm (black circles) and 394 nm (red circles) in 10.0 s. For comparison, the traces at 394 nm (black circles) and 418 nm (red circles) for WT are also shown. WT, wild‐type.

The mutant T310A is then tested for Cpd I formation and Figure 2a shows the spectral changes obtained by mixing the protein with *m*‐CPBA at pH 7.5, 4°C. The spectral transitions observed are similar to those of the WT. Interestingly, the formation and decomposition rates of Cpd I in T310A are faster than those of WT. The absorbance at 380 nm increased more rapidly, reached the maximum in ~0.4 s and then decreased (Figure 2b). By plotting the *k*_obs_ versus *m*‐CPBA concentration, the *k*_1_ of T310A is 0.72 ± 0.03 × 10^5^ M^−1^ s^−1^, which is 2.9 times higher than that of the WT (inset Figure 2a). However, the yield of Cpd I of T310A appears to be the same as WT (Figure 2b). The increased reactivity to *m*‐CPBA may be slightly affected by the increased hydrophobicity near the heme pocket, which makes the binding of *m*‐CPBA more effective.^58^ Also the decomposition rate constant (*k*_2_) increases in the mutant, which is 4.4 times higher than that measured for WT (*k*_2_ = 0.71 ± 0.06 s^−1^ in T310A versus *k*_2_ = 0.16 ± 0.01 s^−1^ in WT), indicating that Cpd I is more unstable for the T310A mutant. When the T310A mutant in complex with the substrate (19‐oxo ASD) is mixed to *m*‐CPBA, a high‐to‐low spin shift similar to the one obtained for WT is detected, indicating substrate turnover and release (Figure 2c,d).

**FIGURE 3:**
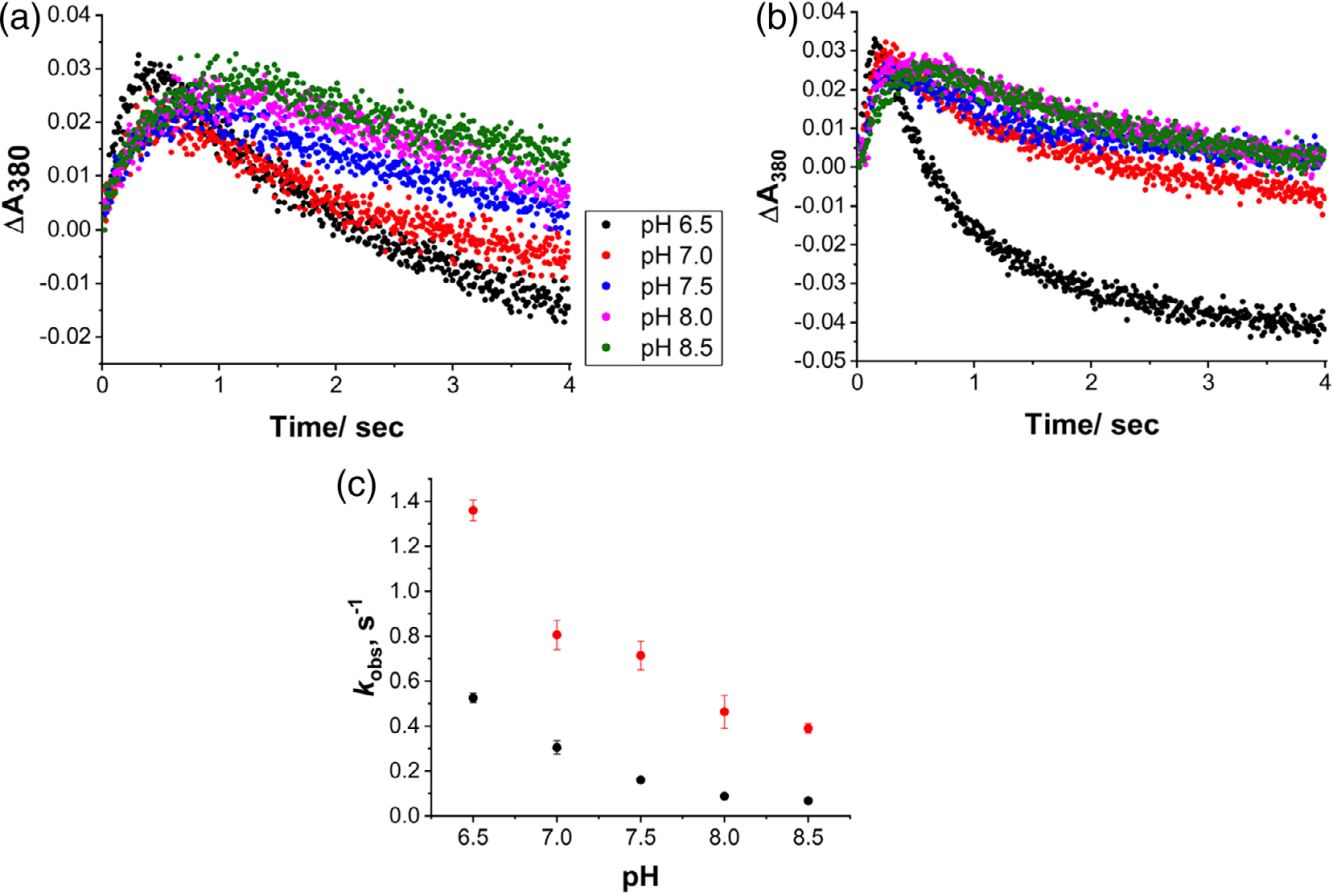
Effect of pH on Compound I formation and decay. (a) Kinetic traces at 380 nm of aromatase WT and (b) T310A at different pH. (c) Plot of the Cpd I decay constant (*k*_2_) obtained for WT (black circles) and T310A (red circles) as a function of pH. WT, wild‐type.

The higher rates of formation of Cpd I in T310A compared to WT are then confirmed at different pH values. Figure 3 shows the time course of formation of Cpd I at 380 nm at different pH. It should be taken into account that the pH dependence of the formation of Cpd I is affected by the pKa of *m*‐CPBA being 7.4.^51^ However, the formation rate decreases significantly from pH 6.5 to 8.5 in both enzymes and the plot of the decay rate *k*_2_ (that is not affected by the pKa of *m*‐CPBA as *k*_1_) against pH shows that the decomposition rate of Cpd I in T310A is higher than that of WT at any pH (Figure 3c), which means that the lifetime of T310A Cpd I is shorter.

It is also interesting to note that the amount of Cpd I is comparable between WT and T310A mutant, indicating that Thr310 is not directly involved in proton relay. Instead, the data indicate T310 has a role in modulating the rate of formation and decay of Cpd I. It is known that the corresponding residue in cytochrome P450cam has a crucial role in dioxygen activation as it accepts a hydrogen bond from the hydroperoxy (Fe(III)‐OOH) intermediate (Cpd 0) promoting the second protonation leading to Cpd I.^38^ Our data show the faster formation and decay rates for Cpd I suggesting that Thr also has a gating role for the second proton transfer as well as a stabilizing role for Cpd I.

### 2.3 Catalytic activity of Aro and mutants at different pH

**FIGURE 4:**
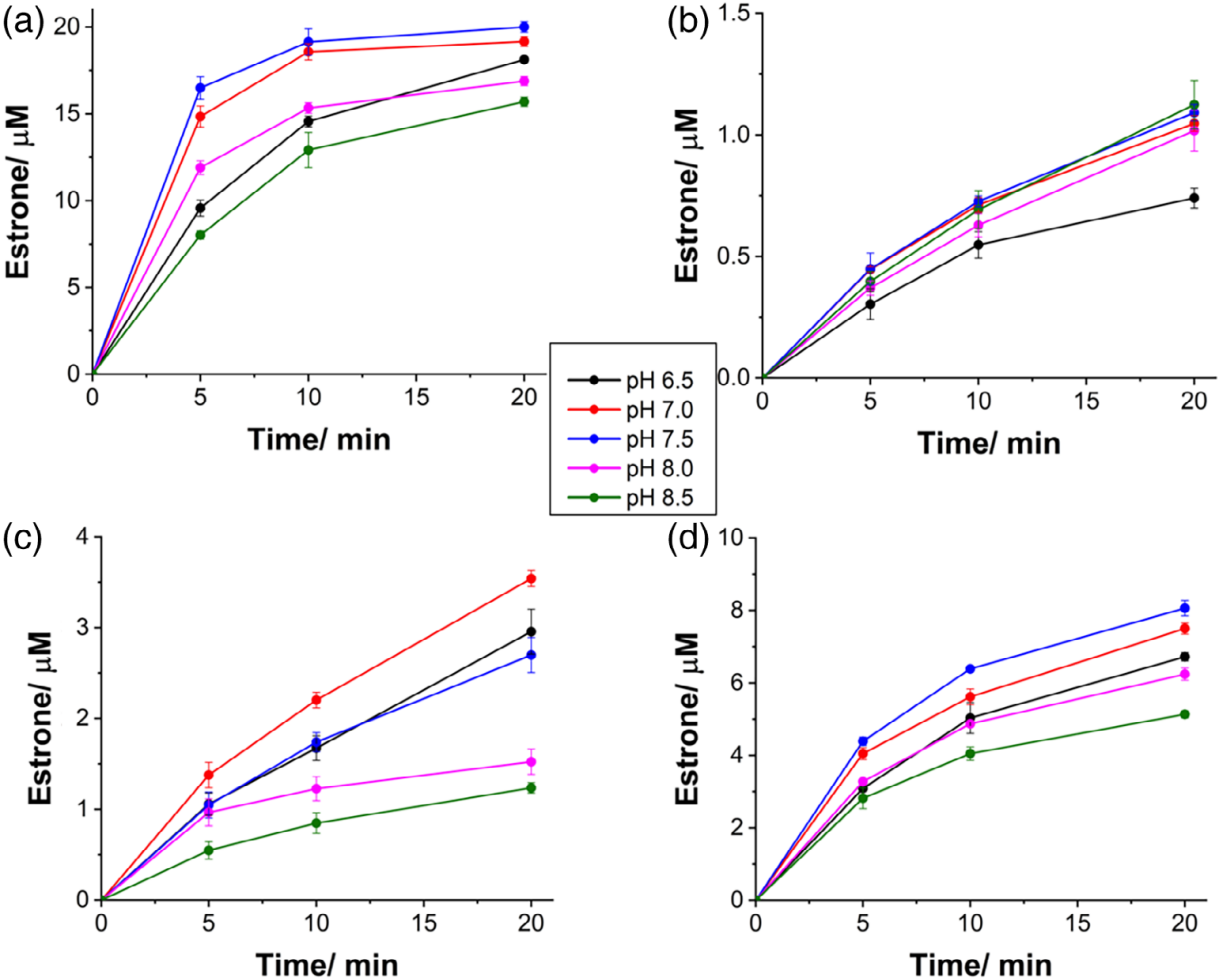
Effect of pH on catalytic activity of WT and its mutants. The activity as a function of time was measured for (a) WT, (b) D309N, (c) R192Q, and (d) T310A. Reaction conditions: 0.5 μM enzyme, 0.5 μM CPR, 20 μM 19‐oxo ASD, 0.5 mM NADPH in 100 mM potassium phosphate buffer, and 10% glycerol, at 30°C. WT, wild‐type.

The next step is to investigate how the different Cpd I formation and decay rates observed for the WT and the mutants at different pH relate to product formation. To correlate the observed different rates of formation and decay of Cpd I at different pH with catalysis in a simplified system, only the third aromatization step is considered. To this end, 19‐oxo ASD is used as the starting substrate and product formation is measured at different pH values. The estrone production is followed as a function of time and the results for the WT and the mutants are shown in Figure 4. As can be seen, the maximal activity in the WT is reached at pH 7.0–7.5 that is reasonable as it corresponds to the physiological pH (Figure 4a). It is interesting to note that the maximal activity is obtained at pH values that do not correspond to the lowest rate of decay of Cpd I, indicating that a higher stability of Cpd I (obtained at pH 8.0–8.5) does not necessarily implicate a higher activity.

In the mutant D309N, the mutation resulted in a dramatic loss of activity by 95% toward 19‐oxo ASD (Figure 4b), consistent with the previous lack of detection of Cpd I. The effect of pH on D309N is lost as the estrone production rate of D309N is almost pH‐independent, consistent with our previous data reporting a pK_a_ of 8.2 for D309 and a crucial role of this residue for catalysis.^35^, ^45^

The mutant R192Q shows a 71% loss of activity compared to the WT. However, a pH dependence of the catalytic activity is present in this mutant and the effect of pH is different compared to WT. Indeed, the maximal activity for this mutant is observed at pH 7.0 (Figure 4c). These data, together with the lack of Cpd I detection previously obtained for this mutant, are consistent with an important role of R192 in proton transfer. However, the residual activity observed in D309N and R192Q mutants together with the different pH dependence of R192Q mutant can be explained the presence of possible alternative but less efficient proton transfer networks in human aromatase.

In T310A mutant, the effect of pH on T310A is similar to that of WT, with the maximal activity observed at pH 7.5 (Figure 4d). Besides, the effect of pH on the catalytic reaction of T310A shows the same trend as that of WT, suggesting that this residue is not responsible for the pH dependence of the catalytic activity in human aromatase. Despite the faster rates of Cpd I formation and decay, the activity is reduced by 60–70% when compared to the WT at every pH value. Thus, this result suggests that a higher formation rate does not necessarily imply a higher activity. The optimal catalytic activity is achieved with intermediate formation and decay rates. Taken together, the data suggest that T310 controls the timing for proton delivery having a proton‐gating role. In addition, it stabilizes Cpd I by optimizing the catalytic activity of human aromatase.

### 2.4 Catalytic rate and uncoupling in WT and mutants

To better understand the role of the three amino acids in proton transfer and catalysis, NADPH consumption, products formation and H_2_O_2_ production are measured. Two sets of experiments are carried out, the first one using androstenedione as the starting substrate to follow the three steps of reaction (Table 1), the second one using 19‐oxoandrostenedione as the starting substrate to follow only the aromatization reaction (Table 1). In both cases, the mutants D309N and R192Q consume a lower amount of NADPH and show to produce a higher amount of H_2_O_2_ compared to WT, consistent with a slower electron‐coupled proton transfer.^59^ The residual activity and the H_2_O_2_ formed indicate that the mutants can still use alternative proton relay networks to deliver the first proton to produce Cpd 0. However, this reactive compound is poorly formed and active as oxidizing species in the three steps of reaction and it decays releasing hydrogen peroxide rather than forming the products increasing the uncoupling of the enzyme.

**TABLE 1:** Rates of NADPH consumption, H_2_O_2_ production, and product formation for Aro and the three mutants

	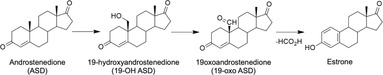							
	NADPH consumption rate (min^−1^)	H_2_O_2_ (−SOD) (min^−1^)	H_2_O_2_ (+SOD) (min^−1^)	19‐OH ASD (min^−1^)	19‐oxo ASD (min^−1^)	Estrone (min^−1^)	Coupling (%)	H_2_O_2_/NADPH (%)
WT	34.6 ± 0.5	2.2 ± 0.2	2.6 ± 0.1	0.48 ± 0.01	0.89 ± 0.02	2.31 ± 0.03	26.6	6.4
D309N	18.6 ± 1.4	3.8 ± 0.2	4.2 ± 0.4	0.06 ± 0.01	0.14 ± 0.01	0.43 ± 0.01	8.8	20.4
R192Q	20.4 ± 2.2	4.1 ± 0.2	5.0 ± 0.3	0.07 ± 0.01	0.23 ± 0.02	0.75 ± 0.01	13.6	20.1
T310A	33.0 ± 0.2	14.2 ± 0.1	15.8 ± 1.3	0.64 ± 0.01	0.27 ± 0.02	0.42 ± 0.01	7.4	43.0

	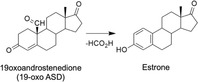							
	NADPH consumption rate (min^−1^)	H_2_O_2_ (−SOD) (min^−1^)	H_2_O_2_ (+SOD) (min^−1^)			Estrone (min^−1^)	Coupling (%)	H_2_O_2_/NADPH (%)
WT	25.1 ± 1.8	1.7 ± 0.3	2.4 ± 0.4			13.1 ± 0.1	52.2	6.8
D309N	18.4 ± 2.5	2.3 ± 0.1	3.1 ± 0.2			0.36 ± 0.01	2.0	12.5
R192Q	22.7 ± 2.2	2.5 ± 0.2	4.1 ± 0.5			1.1 ± 0.1	4.8	11.0
T310A	24.0 ± 2.4	4.2 ± 0.3	5.4 ± 0.2			3.6 ± 0.1	15.0	17.5

On the other hand, the amount of NADPH consumed by WT and T310A mutant are similar, even if the T310A mutant has a lower activity. This is due to an increased amount of H_2_O_2_ produced by the mutant that is 7‐fold higher than the WT when androstenedione is used as substrate.

This result is in line with the corresponding mutants in other P450 enzymes and indicates a lack of stabilization of Cpd 0 by the threonine residue belonging to the acid‐alcohol pair. In the absence of the Thr residue, Cpd 0 is not stabilized by hydrogen bond and it is more easily released as hydrogen peroxide rather than producing Cpd I.^38^

The loss in activity of T310A mutant is not as drastic as in the corresponding mutant in P450cam. Indeed, the mutant shows a 36% residual activity toward androstenedione and a 27.5% residual activity for the aromatization reaction. In this mutant, the amount of Cpd I formed and the NADPH consumption rate are comparable to those of the WT, whereas the amount of H_2_O_2_ is higher. However, the amount of products and H_2_O_2_ formed do not correlate to the level of NADPH consumed indicating that the uncoupling pathway leading to water production from Cpd I is active in the WT and even more so in the T310A mutant. To test this hypothesis, the activity of the enzymes is also measured in the presence of superoxide dismutase (SOD) to check the presence of superoxide anion and no significant increase in the level of H_2_O_2_ is observed. These results again indicate that Thr310 has a stabilizing role not only for Cpd 0 but also for Cpd I. Whether these findings are specific for aromatase is difficult to predict. Indeed, the crystal structure of the enzyme shows a unique proline residue (P308) just before the acidic‐alcohol pair that kinks the helix I and allows the side chain of D309 to form a hydrogen bond with the substrate (Scheme 1c). The oxygen‐binding groove is different when compared to P450cam and the processing of the substrate through a three steps reaction most probably triggers some structural rearrangements involving the active site residues and water molecules. The other major difference is the involvement of the Asp residue in a hydrogen bond with the substrate that is essential for the correct positioning of the substrate in the active site that promotes the highly regioselective hydroxylation at C19. Computational studies have shown that the most energetically favored pathway for aromatase reaction involves substrate enolization as a consequence of proton donation from a water molecule through Asp309. A rotation of this residue induces a water‐mediated proton transfer leading to Cpd I.^46^ Such a mechanism resembles in part what observed in P450cam, where a flip of the Asp residue allows the entry of water molecules and proton transfer.

## 3 DISCUSSION

Cpd I is here captured in aromatase, a human cytochrome P450 that acts as the key player in estrogen production. Its involvement in the third step of reaction is directly demonstrated. By means of site‐directed mutagenesis, the alcohol‐acid pair is shown to play a similar role to the one reported for the bacterial cytochrome P450cam. In aromatase, the aspartic acid residue is involved in proton delivery and its absence almost completely blocks the activity of the enzyme. However, this residue has a double role in aromatase since it was previously demonstrated that it is also responsible for proton delivery to the substrate for aromatization.^45^, ^46^ The protonation‐deprotonation equilibrium of Asp309, allowed by its unusual pK_a_,^45^ is the main responsible for the pH dependence of the catalytic activity in aromatase.

The threonine residue is shown not to be directly involved in proton delivery as Cpd I could be captured for this mutant. However, a proton gating role for this residue and a stabilization effect for Cpd I are shown to have a crucial role in catalysis. The data presented add new insights into the mechanism of dioxygen activation by cytochromes P450.

## 4 MATERIALS AND METHODS

### 4.1 Materials

All chemicals were reagent grade and obtained from commercially available sources. Androstenedione, meta‐chloroperbenzoic acid (*m*‐CPBA), SOD, and nicotinamide adenine dinucleotide phosphate (NADPH) were obtained from Sigma‐Aldrich (Milan, Italy). 19‐oxo androstenedione was purchased from Biozol (Eching, Germany). Amplex Red hydrogen peroxide/peroxidase assay kit was purchased from Invitrogen (Eugene, OR).

### 4.2 Protein expression and purification

The recombinant form of human aromatase (Aro), the mutants (Aro‐D309N, Aro‐T310A, and Aro‐R192Q), and cytochrome P450 reductase (CPR) were expressed and purified as previously described.^55^, ^60^ The P450 enzyme concentration was determined by an Agilent 8453 UV VIS spectrophotometer (Agilent Technologies, Santa Clara, CA) by the reduced carbon monoxide complex in the difference spectrum, using an extinction coefficient at 450 nm of 91,000 M^−1^ cm^−1^.^61^ The CPR concentration was determined by using the extinction coefficient at 456 nm (24,100 M^−1^ cm^−1^).^62^

### 4.3 Cpd I capture and characterization by stopped‐flow kinetics

The compound *m*‐CPBA was purified by washing with 100 mM pH 7.5 potassium phosphate buffer for 3–5 times and stirring on ice for 1 hr. The stock solution of *m*‐CPBA was prepared in acetonitrile and diluted to the appropriate concentration with potassium phosphate (KPi) buffer. The oxidation of ferric aromatase and the mutants was carried out using a Hi‐Tech scientific SF‐61 single mixing stopped‐flow instrument (TgK Scientific, UK). One of the drive syringes was filled with enzyme (14 μM) in a 100 mM potassium phosphate buffer, 10% glycerol at the chosen pH. The second drive syringe was filled with the *m*‐CPBA in the same buffer. All the experiments were carried out at 4°C using a circular water bath. The kinetic data were analyzed with Kinetic Studio software, version 5.1.06 (TgK Scientific, UK).

### 4.4 Activity assay enzyme reaction

For the experiment where *m*‐CPBA was used to form Cpd I and trigger the reaction, a 1 ml solution of 100 mM potassium phosphate buffer, 10% glycerol containing 7 μM aromatase, and 200 μM 19‐oxo‐androstenedione (19‐oxo‐ASD) was incubated at 4°C and titrated with *m*‐CPBA (total addition of 200 μM). The reaction was stopped by heating at 90°C for 10 min. After centrifugation at 11,000*g* for 15 min, the supernatant was used for HPLC analysis. In the control experiment, the enzyme was inactivated by heat shock, and then incubated with *m*‐CPBA.

For the experiment where Aro activity was studied at different pH values, all the reaction mixtures were set up in 100 mM potassium phosphate buffer (at a chosen pH) containing 0.5 μM aromatase, 0.5 μM CPR, 20 μM 19‐oxo‐ASD, and 0.5 mM NADPH at 30°C for 20 min, and the final reaction volume was 0.5 ml. The reaction was stopped by heating at 90°C for 10 min. After centrifugation, the supernatant was further analyzed by HPLC.

### 4.5 NADPH consumption and hydrogen peroxide formation measurement

NADPH consumption was measured by an Agilent 8453 UV–vis spectrophotometer at 30°C. The reaction mixture contained 0.25 μM Aro, 0.25 μM CPR, 100 μM substrate androstenedione (ASD) or 19‐oxo‐androstenedione (19‐oxo ASD), 150 μM NADPH, in a volume of 400 μl. After adding NADPH, the absorbance of the reaction solution at 340 nm was recorded every 20 s for 10 min. The NADPH consumed was calculated by using an extinction coefficient at 340 nm of 6,220 M^−1^ cm^−1^ for NADPH. The products were quantified by HPLC analysis.

The concentration of hydrogen peroxide generated during NADPH consumption was determined by Amplex Red hydrogen peroxide/peroxidase assay kit according to the manufacturer's instructions. The superoxide radical was determined by adding 2 μM SOD under the same conditions.

### 4.6 HPLC analysis

The HPLC analysis was performed on an Agilent 1200 series HPLC system (Agilent Technologies, Santa Clara, CA) equipped with a diode array detector (DAD) and a reverse‐phase column (Zorbax Eclipse plus C18, 250 × 4.6 mm^2^, 5 μm, Agilent Technologies, Santa Clara, CA). A sample volume of 85 μl was injected into the HPLC system for each analysis.

When 19‐oxo ASD was used as the substrate, the mobile phase was water: acetonitrile (50:50, V/V), the flow rate was 0.5 ml/min, and the wavelength set at 280 nm to detect estrone.

When ASD was used as the substrate, the reaction solution was analyzed by the following program, using water and acetonitrile as mobile phase: 0–3 min, 5% acetonitrile; 3–18 min, linear gradient from 5 to 50% acetonitrile; 18–23 min, linear gradient to 100% acetonitrile; 23–32 min, isocratic at 100% acetonitrile. The flow rate was 0.5 ml/min. In this case, ASD, 19‐OH ASD, and 19‐oxo ASD were detected at 237 nm, and estrone was detected at 280 nm.

## AUTHOR CONTRIBUTIONS

**Chao Zhang:** Data curation (equal); formal analysis (equal); methodology (lead); software (lead); validation (equal); visualization (equal); writing – original draft (equal). **Gianfranco Gilardi:** Conceptualization (equal); formal analysis (equal); funding acquisition (equal); investigation (equal); writing – review and editing (equal). **Giovanna Di Nardo:** Conceptualization (equal); data curation (equal); formal analysis (equal); funding acquisition (equal); methodology (equal); project administration (lead); supervision (lead); writing – original draft (equal); writing – review and editing (equal).

## CONFLICT OF INTERESTS

The authors declare no conflicts of interest.

## Supporting information


**FIGURE S1.** HPLC traces showed the *m*‐CPBA‐driven formation of estrone product. (a) The chromatographic profiles obtained from the control. (b) The estrone standard. (c) The reaction mixture. The reaction mixture contained 7 μM of Aro, 200 μM *m*‐CPBA, 200 μM 19‐oxo ASD in 100 mM potassium phosphate buffer (10% glycerol) at pH 7.5 and 4°C. The control reaction contained heat‐inactivated Aro. The analyses were performed at a flow rate of 0.5 ml/min of water/acetonitrile (50/50, v/v). The detection wavelength was 280 nm.
**FIGURE S2**. *m*‐CPBA reacted with mutants in the presence or absence of substrate. (a) Substrate‐free low‐spin ferric D309N. Scanning time, 4 s. (b) Substrate‐bound high‐spin ferric D309N. Scanning time, 400 s. (c) Substrate‐free low‐spin ferric R192Q. Scanning time, 4 s. (d) Substrate‐bound low‐spin ferric R192Q. Scanning time, 40 s. Reaction condition: enzyme was mixed 1:1 (v/v) with 25 eq mol of *m*‐CPBA in 100 mM potassium phosphate buffer, 10% glycerol, pH 7.5, at 4°C.
