# Supplementary material for: Depicting the proton relay network in human aromatase: New insights into the role of the alcohol‐acid pair
Source: Protein Sci. 2022 Aug 11;31(9):e4389. doi: 10.1002/pro.4389 (PMC9366932; doi:10.1002/pro.4389)
Supplement: Supplementary file 1 — FIGURE S1. HPLC traces showed the m‐CPBA‐driven formation of estrone product. (a) The chromatographic profiles obtained from the control. (b) The estrone standard. (c) The reaction mixture. The reaction mixture contained 7 μM of Aro, 200 μM m‐CPBA, 200 μM 19‐oxo ASD in 100 mM potassium phosphate buffer (10% glycerol) at pH 7.5 and 4°C. The control reaction contained heat‐inactivated Aro. The analyses were performed at a flow rate of 0.5 ml/min of water/acetonitrile (50/50, v/v). The detection wavelength was 280 nm. FIGURE S2. m‐CPBA reacted with mutants in the presence or absence of substrate. (a) Substrate‐free low‐spin ferric D309N. Scanning time, 4 s. (b) Substrate‐bound high‐spin ferric D309N. Scanning time, 400 s. (c) Substrate‐free low‐spin ferric R192Q. Scanning time, 4 s. (d) Substrate‐bound low‐spin ferric R192Q. Scanning time, 40 s. Reaction condition: enzyme was mixed 1:1 (v/v) with 25 eq mol of m‐CPBA in 100 mM potassium phosphate buffer, 10% glycerol, pH 7.5, at 4°C. [file PRO-31-e4389-s001.pdf]

Supporting Information for

Depicting the proton relay network in human aromatase: new insights into the role of the alcohol-acid pair

Chao Zhang, Gianfranco Gilardi* and Giovanna Di Nardo*

Department of Life Sciences and Systems Biology, via Accademia Albertina 13, 10123, Torino, Italy

Table of contents

Figure S1 3
Figure S2 4

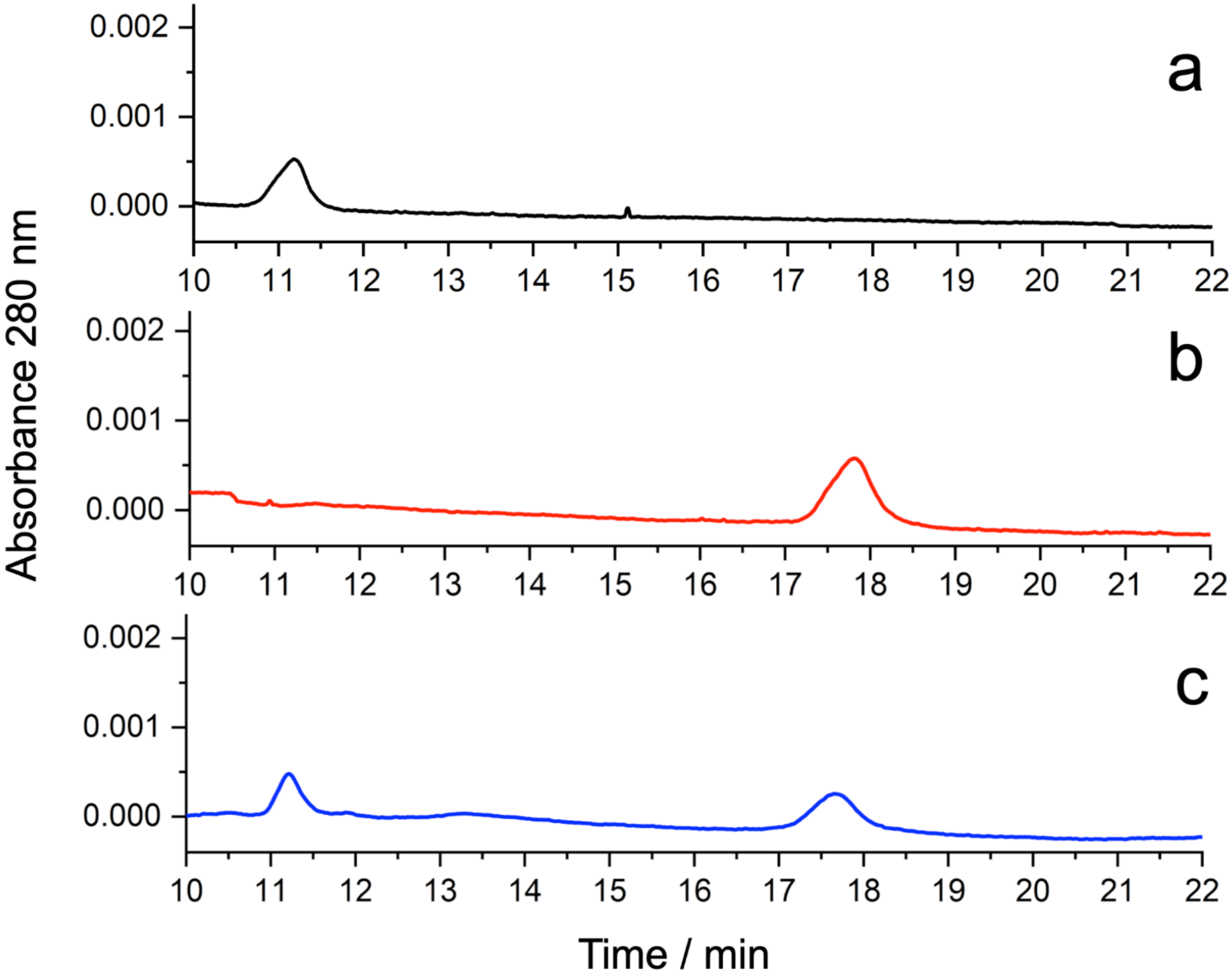

Figure S1. HPLC traces showed the *m*-CPBA-driven formation of estrone product. (a) The chromatographic profiles obtained from the control. (b) The estrone standard. (c) The reaction mixture. The reaction mixture contained 7 μM of Aro, 200 μM m-CPBA, 200 μM 19-oxo ASD in 100 mM potassium phosphate buffer (10% glycerol) at pH 7.5 and 4 °C. The control reaction contained heat-inactivated Aro. The analyses were performed at a flow rate of 0.5 mL/min of water/acetonitrile (50/50, v/v). The detection wavelength was 280 nm.

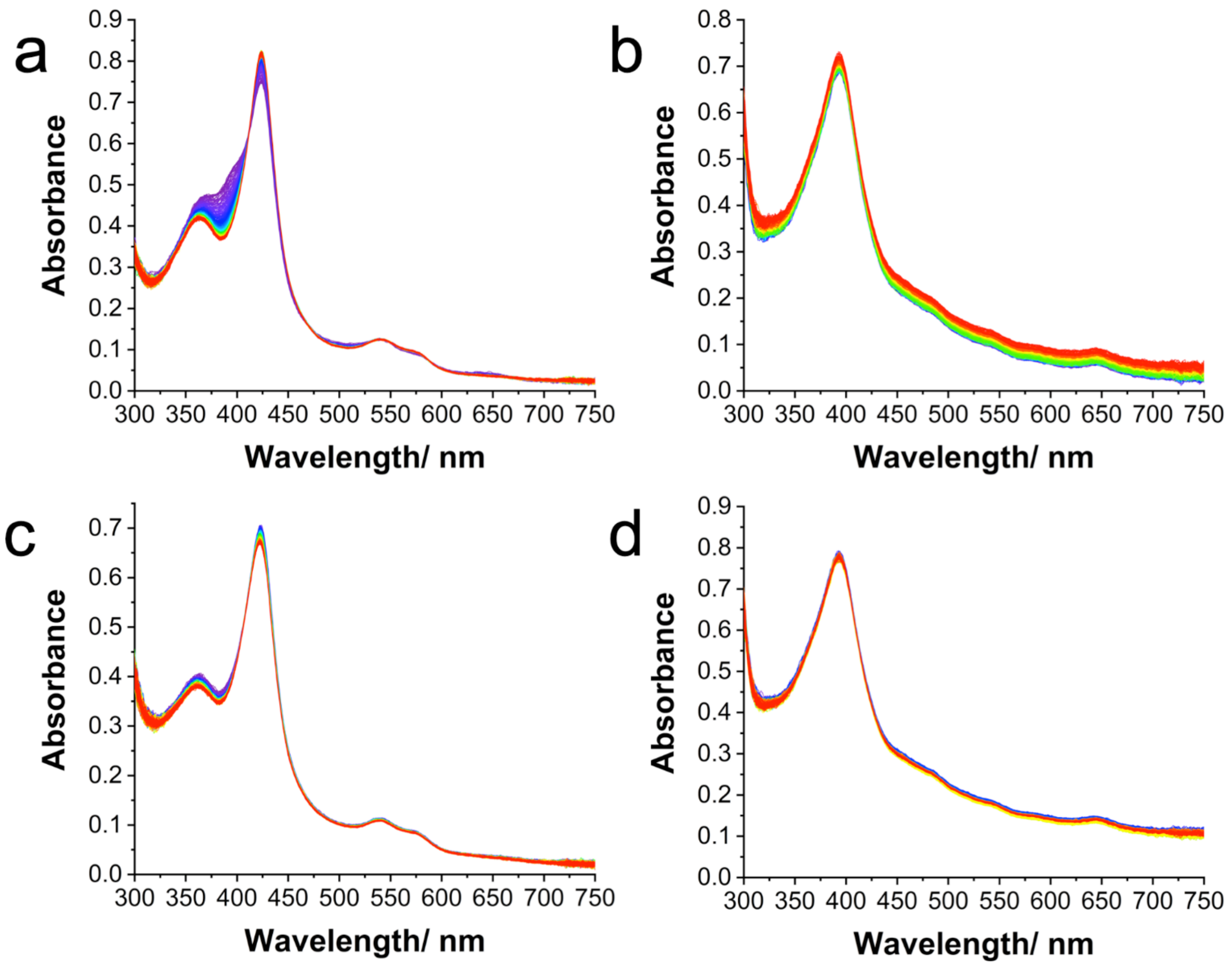

Figure S2. *m*-CPBA reacted with mutants in the presence or absence of substrate. (a) Substrate-free low-spin ferric D309N. Scanning time, 4 s. (b) Substrate-bound high-spin ferric D309N. Scanning time, 400 s. (c) Substrate-free low-spin ferric R192Q. Scanning time, 4 s. (d) Substrate-bound low-spin ferric R192Q. Scanning time, 40 s. Reaction condition: enzyme was mixed 1:1 (v/v) with 25 eq mol of *m*-CPBA in 100 mM potassium phosphate buffer, 10% glycerol, pH 7.5, at 4 ° C.
